# Nanotechnology-Based Detection of Sickle Cell Disease and Thalassemia: A Systematic Review

**DOI:** 10.3390/bios16070373

**Published:** 2026-07-08

**Authors:** Manjyot Kaur, Janesh Kumar Gautam, Aishwarya Rajendra Sharma, Vishal Singh, Disha Chouhan, Akash Baghel, Bontha V. Babu, Suman Sundar Mohanty

**Affiliations:** 1 ICMR-National Institute for Implementation Research on Non-Communicable Diseases, New Pali Road, Jodhpur 342005, India; manjyotkaur31@gmail.com (M.K.); janeshgautam@gmail.com (J.K.G.); aishwaryasharma0598@gmail.com (A.R.S.); dishachouhan1020@gmail.com (D.C.); im.akashbaghel@gmail.com (A.B.); babubontha.hq@icmr.gov.in (B.V.B.); 2 Department of Neurology, The College of Medicine, The Ohio State University, Columbus, OH 43210, USA; sin218@osumc.edu

**Keywords:** sickle cell disease, thalassemia, hemoglobinopathy, nanotechnology, biosensor

## Abstract

Sickle cell disease (SCD) and thalassemia are genetic disorders that necessitate accurate diagnosis for effective management and improved patient outcomes. The advent of nanotechnology has paved the way for innovative, precise detection methods, offering enhanced sensitivity and specificity. The present systematic review aims to assess the analytical performance of nanotechnology-based detection methods for SCD and thalassemia, with a focus on evaluating the analytical performance and identifying the most sensitive nanotechnology-based techniques. An extensive literature search was conducted across five databases (ScienceDirect, PubMed, Embase, Google Scholar), yielding 23 studies that met the inclusion criteria. These studies showcased the potential of nanotechnology-based methods for detecting SCD and thalassemia. The studies utilized diverse samples, including blood, serum, genomic DNA, and purchased oligonucleotides, with most reporting limit of detection (LOD) values. Specifically, gold nanoparticles (AuNPs) exhibited exceptional sensitivity, with detection limits ranging from 2.6 aM to 0.035 pM. Surface modification and functionalization of AuNPs significantly enhance their detection capabilities. Other nanostructures, including silver nanoparticles, quantum dots, and graphene quantum dots, also demonstrate promising diagnostic capabilities. The results showed that nanotechnology-based methods demonstrated improved analytical sensitivity, with LOD ranging from 2.6 aM to 50 nM. This systematic review provides a comprehensive overview of the analytical performance of nanotechnology-based detection methods, shedding light on their potential to revolutionize diagnosis and treatment. Overall, it highlights the transformative potential of nanotechnology in improving molecular diagnostic accuracy for SCD and thalassemia.

## 1. Introduction

Sickle cell disease (SCD) and thalassemia are genetic disorders affecting millions worldwide, posing significant diagnostic and therapeutic challenges despite advances in medical science [1,2]. SCD predominantly affects the populations from sub-Saharan Africa, the Mediterranean, Latin America, and the Middle East, India, while thalassemia affects over 280 million people worldwide [3]. The mutations affect the β-globin chain of hemoglobin, which causes hemoglobinopathies, including SCD. SCD causes chronic hemolytic anemia, severe pain, and end-organ damage [4,5,6]. Thalassemia, a disorder affecting hemoglobin production, is characterized by defective alpha or beta chains of hemoglobin due to genetic mutation [7,8,9,10], which lead to anemia, fatigue, and other complications [11,12,13,14]. To date, over 250 mutations have been found to be responsible for beta-thalassemia, including point mutations and larger deletions [8,10,14,15]. Defects at genetic and molecular levels cause abnormal genetic expression, leading to the abnormal production of beta-globin chains and the development of beta-thalassemia [15,16,17]. Prevention and early diagnosis of disease complications along with the management of end-organ damage are crucial for improving patient outcomes in both diseases [18].

The clinical significance of early diagnosis and treatment for SCD and thalassemia cannot be overstated. Timely intervention can substantially enhance life quality, thereby reducing the incidence of morbidity and mortality. However, traditional diagnostic methods, such as hemoglobin electrophoresis and high-performance liquid chromatography (HPLC), have limitations, including cost, accessibility, and requirement of technical expertise [19,20,21]. Recent advances in nanotechnology have improved diagnostic accuracy and sensitivity for various diseases [22]. Nanomaterials (NMs) with dimensions less than 100 nm exhibit unique physicochemical properties because of their increased surface-to-volume ratio. This property makes them highly susceptible to external influences, setting them apart from their bulk counterparts [23,24,25,26]. The composition, morphology, and size of NMs can be tailored for specialized applications, such as the creation of nanostructures with properties or various surface functionalities suitable for biosensing. The integration of NMs into detection methods enhances their performance by amplifying signals, thereby increasing detection sensitivity. In cancer diagnostics, nano-biosensors have enabled the non-invasive detection of biomarkers and real-time monitoring of cancer growth [27,28]. Nanoparticles (NPs) and nanosensors have also shown promise in detecting anemia, blood cancer, and bleeding disorders. These technologies aim to create diagnostic tools that are effective, cost-efficient, and highly sensitive [29].

Researchers have explored various nanostructures (silver, gold, graphene, silica, and quantum materials) for diagnosing and treating conditions like hemoglobin disorders [30]. Nanotechnology-based diagnostics offer potential benefits for SCD and thalassemia. Despite these advancements, the application of nanotechnology in diagnostics of these genetic disorders is still in its early stages. This systematic review delves into the existing evidence on the analytical performance of nanotechnology-based advances for the diagnosis of these disorders, evaluating their limitations and potential applications. The included studies provide valuable insights into the development of effective diagnostics, identify areas for future research, and ultimately contribute to improving patient outcomes and quality of life. Thus, the objective is to systematically identify and evaluate nanotechnology-based approaches for detecting SCD and thalassemia, with a focus on their analytical performance and analyte/sample requirements.

Several reviews have addressed nanotechnology-based biosensing approaches for genetic disorders and hemoglobinopathies. Early reviews primarily discussed the types of nanomaterials (e.g., gold nanoparticles, quantum dots, graphene) and their role in enhancing biosensor performance [19,30]. Recent works have focused on nanotechnology-enabled biosensors, their fabrications, architectural design [31], and their substantial medical applications in the detection of various diseases like Diabetes [32], Parkinson’s disease [33], and Glaucoma [34]. These studies mainly focus on underlying principles, applications, and limitations of advancing biosensors in medical applications [32,33]. Few studies have also discussed the role of nano-biosensors for point-of-care genetic diagnostics [35], and summarized its potential in the development of various theragnostic approaches to determine thalassemia-causing gene mutations [36].

While these reviews provide valuable insights into nanotechnology-enabled diagnostics, most emphasize either material types or general sensing strategies, with limited comparison of analytical performance metrics (LOD, linear ranges), functionalization approaches, and the extent of clinical validation. In contrast, our review systematically compiles nanotechnology-based diagnostic methods for sickle cell disease and thalassemia, with an emphasis on analytical performance, translational readiness, and limitations, thereby addressing an important gap in the current literature. The current review work advances beyond prior descriptive surveys to provide a performance-based framework that identifies the most promising nanoplatforms and clarifies the translational hurdles that must be addressed.

## 2. Materials and Methods

### 2.1. Focus Question

Which nanotechnology-based approaches/techniques have been explored for the detection of SCD and thalassemia, and what are their respective LOD and sample/analyte requirements? (Table 1).

### 2.2. Inclusion Criteria

1. Study Design: Original research articles, including experimental, observational, and diagnostic accuracy studies.
2. Population: Studies focusing on human subjects with SCD and thalassemia.
3. Intervention/Exposure: Studies evaluating the use of nanotechnology for the detection or quantification of SCD and thalassemia.
4. Outcomes: Studies reporting on analytical sensitivity, analytical specificity, limit of detection and limit of quantitation.
5. Language: Studies published in English.
6. Publication Status: Peer-reviewed articles published in scientific journals.
7. Timeframe: Studies published from inception to the present.

### 2.3. Exclusion Criteria

1. Editorials, or conference proceedings
2. Studies focusing on non-human subjects
3. Studies not using nanotechnology
4. Abstracts or unpublished data
5. Review or systematic review articles.
6. Studies published in languages other than English.

### 2.4. Search Strategy and Information Sources

The Preferred Reporting Items for Systematic Review (PRISMA) guidelines were followed for this systematic review, with protocol registration on PROSPERO (CRD42025631798) and compliance with PRISMA 2020 checklist for study selection, screening, analysis, and reporting.

1. Preliminary search: A limited search of PubMed to identify relevant articles.
2. Keyword extraction: Relevant words from article titles, abstracts, and index terms were extracted to develop a full search strategy.
3. Database searching: Multiple databases/sources (listed in Table 2) were searched using the adapted search strategy.
4. Vocabulary control: Controlled vocabularies were used to identify synonyms and index terms.

### 2.5. Study Selection and Screening Process

The present systematic review process began with the removal of duplicate studies from the initial pool, and Rayyan was used to streamline this process. The titles and abstracts of the remaining studies were carefully screened by three independent reviewers. The studies were initially screened based on predefined inclusion and exclusion criteria. Selected studies were then retrieved for full-text review. Methodology, results, and conclusions of the study were thoroughly evaluated. If a study was excluded during the full-text review, the reason for its exclusion was documented. In cases where the two reviewers disagreed, the issue was resolved through discussion or consultation with the third expert.

### 2.6. Data Extraction

Data from all the selected studies was extracted, such as title, name of journal, year of publication, country, background, objective, study design, nanotechnology used, modifications with nanomaterials, samples used, number of subjects, techniques used for detection, and key findings. Analytical outcomes such as sensitivity, specificity, LOD, and LOQ were also extracted.

### 2.7. Data Synthesis

Considering the diverse nature of the data obtained from the included studies, conducting a meta-analysis was deemed unfeasible. Alternatively, a narrative synthesis approach was used to summarize and interpret the findings, which are discussed in the result section. This approach sought to provide a comprehensive overview of the included studies, including their characteristics, methodologies, and outcomes.

## 3. Results and Discussion

### 3.1. Study Selection

**Figure 1:**
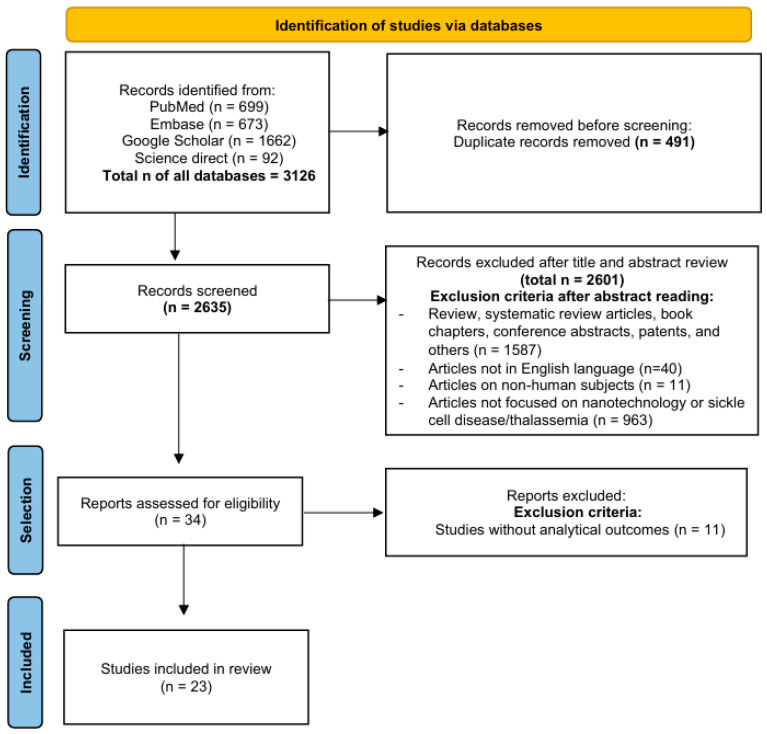
PRISMA flow diagram: Study selection process for systematic review. This figure illustrates the systematic process used to select studies for inclusion in this review, adhering to the PRISMA guidelines.

This comprehensive literature search yielded a substantial number (3126) of studies across various databases. Specifically, 699 studies on PubMed, 1662 on Google Scholar, 673 on Embase, and 92 on ScienceDirect were found. After removing duplicate or triplicate entries, a meticulous screening of the title and abstract was conducted, adhering to predefined inclusion and exclusion criteria (in Section 2.2 and Section 2.3). This rigorous selection process led to the identification of 23 studies that ultimately met the priority criteria, focusing on the application of nanotechnology for the detection of SCD and thalassemia (Figure 1).

### 3.2. Analytical Performance of Nanotechnology-Based Approaches

**Figure 2:**
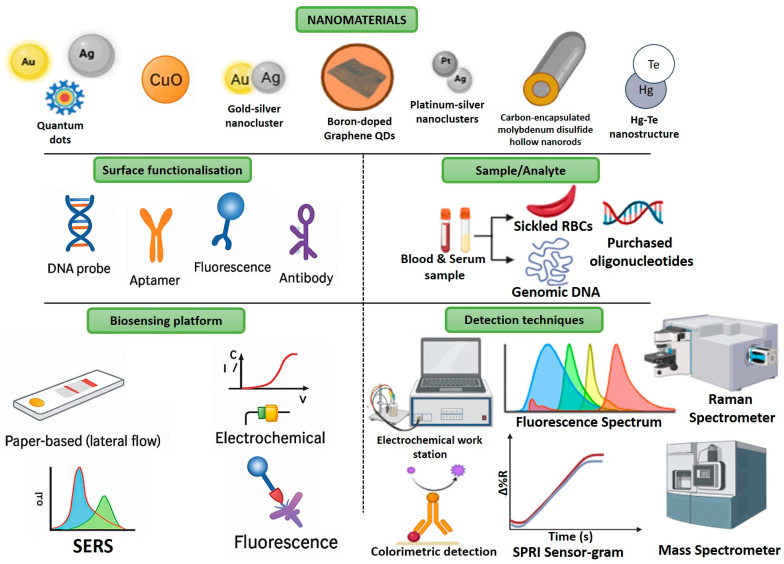
A simple illustration of various NPs, sample requirements and detection techniques being used for SCD and thalassemia nanodiagnostics. (Abbreviations: Au—gold; Ag—silver; CuO—copper oxide, Au-Ag—gold–silver nanocluster; QDs—quantum dots; Pt-Ag—platinum–silver nanoclusters; Hg-Te—mercury telluride; SPRI—surface plasmon resonance imaging, SERS—surface enhanced Raman scattering).

This review highlights the analytical performance of various nanotechnology-based approaches in detecting SCD and Thalassemia. Nanostructured materials, including metal nanomaterials, quantum dots (QDs), graphene oxide, and magnetic nanoparticles (MNPs) have been successfully applied in the detection of SCD and thalassemia (Figure 2) in biological samples such as blood, serum, and DNA (Table 3, Table 4, Table 5 and Table 6). The detection limits of various nanotechnology-based approaches for detecting SCD and thalassemia have been a pivotal aspect of research in this domain. According to the literature, reported LOD varies widely, ranging from a few attomolar to several nanomolar concentrations.

#### 3.2.1. Gold Nanoparticles

Gold nanoparticles (AuNPs) have emerged as the most commonly used NP in detecting SCD and thalassemia. The detection limits of AuNP-based approaches range from 2.6 aM to several pM [39,40,49]. A study reported a detection limit of 5.2 × 10^−16^ M for the fetal β-thalassemia gene in blood samples of pregnant mothers [40]. Another study reported LOD of 0.571 pM and ability to discriminate between heterozygous and homozygous β-thalassemia genetic disorders [45]. Similarly, a recent study aimed to detect point mutations directly in non-amplified human genomic DNA using a AuNPs-enhanced surface plasmon resonance imaging (SPRI) platform [39]. The assay could discriminate between healthy, heterozygous, and homozygous β-thalassemia genotypes and achieved detection sensitivity of 2.6 aM [39]. However, it lacks large-scale clinical validation as the study consisted of a total of 18 samples, including genomic DNA from 18 healthy individuals, 7 homozygous, and 7 heterozygous β-thalassemia patients [39].

Another study evaluated detection limits for CD122, a gene associated with thalassemia, by utilizing AuNPs (LOD = 78.7 aM) and carbon-encapsulated molybdenum disulfide hollow nanorods (MoS_2_@C) (LOD = 58.5 aM) [49]. Gold nanocomplexes like gold–silver bimetallic (Au@Ag) NPs, AuNPs combined with graphene oxide NPs and AuNPs/GDY nanocomposite, have shown promising detection limits of 5.13 fM—0.0481 pM for β-thalassemia [50,51,57] and 40 pM for sickle cell anemia (SCA) [52], respectively. The current study aimed at detecting microRNA-210 (miR-210), a biomarker linked to γ-globin gene regulation in β-thalassemia using Au@Ag NPs and surface-enhanced Raman scattering (SERS) [50]. This biosensor demonstrated LOD of 5.13 fM, validated using erythrocytes samples (*n* = 8) from healthy (*n* = 4) and β-thalassemia patients (*n* = 4). While promising for rapid miR-210 detection in β-thalassemia, broader clinical validation is needed to confirm its utility in diverse patient populations and routine diagnostic settings [50].

These findings indicate AuNPs as one of the most sensitive nanomaterials for detecting SCD and thalassemia, outperforming other nanoplatforms like quantum dots, silver nanoparticles, and boron-doped graphene, having higher detection limits.

#### 3.2.2. Quantum Dots

In recent years quantum dots have been explored as a potential alternative to AuNPs because of their enormous potential in ultrasensitive analysis [47]. A study evaluated the quantification limit (0.27 ng) of quantum dots for detecting ferritin levels in blood serum samples [47]. This study was validated across multiple patient (*n* = 16) sera groups, including thalassemia patients (*n* = 4), hepatoma patients (*n* = 10), and normal individuals (*n* = 2) [47]. Although this detection limit is higher than that reported for AuNPs, quantum dots have shown promise in detecting protein biomarkers associated with thalassemia. Boron-doped graphene quantum dots have been reported to have a detection limit of ∼(0.005 ± 0.001) μM for detecting hematin levels in sickle erythrocytes. The primary focus is on developing a method for detecting hematin, not specifically for detecting SCD itself [59]. This study is a type of analytical proof-of-concept, with a total of seven samples, five healthy and two SCD samples. However, given the limited patient sample set, further validation with larger, diverse cohorts and inclusion of standard-method comparisons will be crucial for clinical translation [59]. Such limited cohorts increase the risk of selection bias, lack of statistical power, and overestimation of diagnostic performance. The restricted patient diversity in these studies also raises concerns regarding reproducibility and generalizability across broader populations. Nevertheless, the method could potentially be used as a diagnostic tool or biomarker for SCD, as the study shows that hematin levels are significantly higher in sickle cell erythrocytes compared to healthy ones [59]. Quantum dots exhibit comparatively higher detection limits than AuNPs, although their ability to detect specific protein and metabolic biomarkers still holds significant diagnostic value. Their tuneable optical properties and potential for multiplexing make them a viable alternative in targeted applications.

While these findings highlight the diagnostic potential of quantum dots, the very small sample sizes represent a major limitation. Therefore, although quantum dots demonstrate promising analytical capabilities, large-scale validation in diverse clinical cohorts is urgently needed before these approaches can be translated into routine diagnostics.

#### 3.2.3. Silver Nanoparticles

Silver nanoparticles (AgNPs) have also emerged as a valuable tool for detecting SCD and thalassemia. A recent study utilized AgNPs for detection of single nucleotide polymorphism (SNP) to identify sickle cell anemia [46]. Results indicated a detection limit of 10^3^ copies per μL of SNP targets in serum [46]. Although this detection limit is higher than that of AuNPs, AgNPs have demonstrated remarkable potential in identifying genetic mutations linked to SCD. Furthermore, the combination of platinum and AgNPs has shown promise in detecting these mutations and achieving a detection limit of 470.0 pg/μL [53]. Similarly, the incorporation of platinum into AgNPs, forming Ag/Pt bimetallic nanoclusters, has significantly enhanced detection capabilities, achieving an impressive detection limit of 0.8 fM for single-nucleotide variants related to β-thalassemia [55]. Despite having relatively higher detection limits, silver-based nanostructures have demonstrated enhanced sensitivity and specificity in detecting hemoglobinopathies, which underscores their potential in advanced molecular diagnostics.

#### 3.2.4. Other Nanoparticles

**Figure 3:**
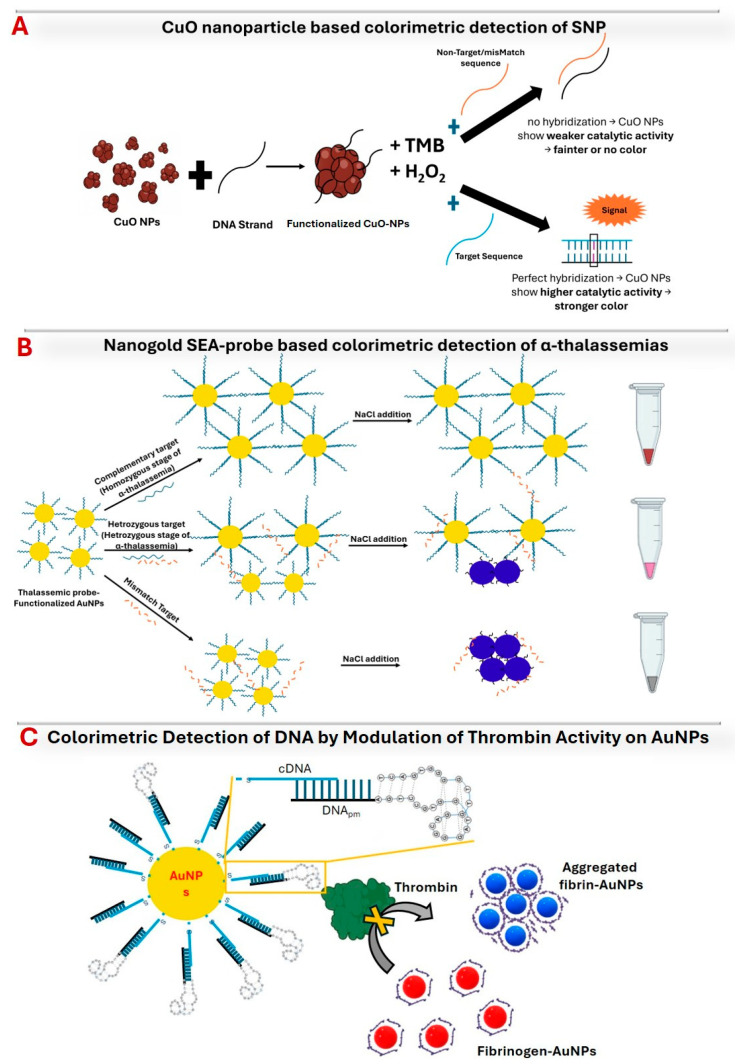
Schematic illustration of colorimetric-based detection of SCD/thalassemia. (**A**) CuO NPs-based colorimetric detection of SNP; adapted with modifications from [48]. (**B**) Nanogold SEA-probe-based colorimetric detection of α-thalassemia; adapted with modifications from [43]. (**C**) Colorimetric detection of SCA-related SNP using thrombin-binding aptamer (hTBA29)–AuNPs; adapted with modifications from [38]. (Abbreviations: CuO—copper oxide; NPs—nanoparticles; TMB—tetramethylbenzidine; H_2_O_2_—hydrogen peroxide; NaCl—sodium chloride; AuNPs—gold nanoparticles; cDNA—complimentary DNA).

Even so, less commonly used, other NPs like magnetic, copper oxide, and upconversion NPs have also demonstrated commendable sensitivity in detecting hemoglobinopathies [30,48,50]. A study developed an oligonucleotide sensor using photon upconverting NPs (NaYF_4_ doped with Yb^3+^ and Er^3+^ for the detection of point mutation associated with SCD). The detection limit of this sensor was found to be 120 femtomoles [56]. Another study established a colorimetric nanobiosensor by utilizing copper oxide (CuO) NPs to detect SNP in cell-free fetal DNA. This nanobiosensor detected the target sequence even with one nucleotide polymorphism with a detection limit of 0.64 nM [48]. Similarly, mercury telluride (HgTe) was used as the matrix for the detection of SNP in sickle cell megaloblasts, with a detection limit of 0.05 μM [54]. In a different study, magnetic NPs were utilized to quantify hepcidin levels, a key regulator of mammalian iron metabolism, and the lower limit of quantitation was found to be 0.4 nmol/L [58]. The unique physicochemical properties of these other NPs offer valuable alternatives for targeted biomarker detection in hemoglobinopathies. Figure 3 summarizes the schematic illustration of different colorimetric-based detection methods reported for SCD/thalassemia. Figure 3A depicts CuO NPs-based colorimetric detection of SNP. This biosensor works on the peroxidase-like activity of CuO NPs, which becomes stronger when bound to double-stranded DNA. This enhanced activity oxidizes TMB in the presence of H_2_O_2_, producing a visible color change that allows detection of SNPs related to SCA [48]. Similarly, Figure 3B shows nanogold SEA-probe-based colorimetric detection of α-thalassemia. This detection method is based on the salt-induced aggregation of AuNPs, as in the presence of complementary target DNA, AuNP–probe conjugates remain dispersed even after NaCl addition, showing a red solution. Absence of hybridization leads to salt-induced aggregation and a blue solution, while partial hybridization in carriers results in a purple color [43]. Another interesting work on the colorimetric detection of SCA-related SNP is represented in Figure 3C. The AuNPs are probed with thrombin-binding aptamer (hTBA29)–AuNPs. If the DNA matches, then it inhibits thrombin and maintains dispersed red AuNPs, while mismatched/absent DNA allows thrombin activity, causing fibrinogen–AuNP aggregation and a blue color shift [38].

#### 3.2.5. Sample/Analyte Requirements

The detection of various analytes associated with SCD and thalassemia has been reported using nanotechnology-based approaches. These analytes can be broadly categorized into several groups, including specific DNA sequences, microRNAs, genomic DNA, cell-free fetal DNA, proteins, and SNPs. Specific DNA sequences, such as the β-globin gene [37,39,49], CD122 gene [49], CD142 gene [51], and oligonucleotides related to α thalassemia [43] and β-thalassemia [42,44], have been detected using nanotechnology-based approaches. These DNA sequences are associated with various aspects of SCD and thalassemia, including disease diagnosis, monitoring, and treatment. MicroRNAs, such as microRNA-21035, have also been detected using these approaches. MicroRNAs play a crucial role in controlling gene expression, and their detection can provide valuable information about the underlying molecular mechanisms of SCD and thalassemia. Genomic DNA and cell-free fetal DNA were likewise detected using nanotechnology-based approaches, demonstrating their capability for analyzing complex genomic samples and non-invasive prenatal diagnosis [39,48]. These approaches have the potential to revolutionize the field of genetic testing and prenatal diagnosis. Similarly, human serum ferritin has been detected using quantum dots, highlighting the potential of nanotechnology-based approaches for identifying protein biomarkers. Ferritin is an important protein biomarker for iron deficiency and overload, which are common complications in SCD and thalassemia [49]. SNPs related to SCD and β-thalassemia have also been detected using nanotechnology-based approaches [38,55]. SNPs are genetic variations that can influence an individual’s risk of developing a disease, and their detection can provide valuable insights into the molecular mechanisms underlying SCD and thalassemia. Overall, the diversity of analytes that can be detected using nanotechnology-based approaches highlights their potential for diagnosing and monitoring SCD and thalassemia. The wide range of sample types, volumes, and target analytes further underscores the versatility and practicality of nanotechnology-based diagnostic approaches.

It is important to note that while some studies reported exceptionally low LOD values (in the attomolar to femtomolar range), many of these were achieved using synthetic oligonucleotides or laboratory-prepared DNA targets [39,49,51,55]. Only a subset of assays demonstrated comparable sensitivity in patient-derived samples, such as the AuNP-based SPRI assay for β-thalassemia [39], Au@Ag nanoparticle-based SERS assay for miR-210 [50], and quantum dot-based ferritin detection [47]. This distinction underscores the gap between laboratory proof-of-concept sensitivity and real-world clinical performance, where complex matrices, variable biomarker abundance, and sample preparation steps can influence assay reliability. Thus, the ultralow LODs reported should be interpreted as indicators of analytical potential rather than direct measures of validated clinical sensitivity.

To address concerns regarding clinical applicability, we summarized in Table 3, Table 4 and Table 5 whether the reported LOD values were obtained from oligonucleotides or human samples. As shown, several ultralow detection limits (attomolar to femtomolar) were achieved only under controlled conditions with synthetic targets [49,51,54,55]. By contrast, studies using patient-derived samples—such as AuNP-SPRI for β-thalassemia [39], Au@Ag SERS assay for miR-210 [50], and quantum dot ferritin detection [47]—reported slightly higher but clinically relevant LODs. This distinction highlights that while nanoplatforms demonstrate remarkable analytical potential, their translation to clinical practice requires validation in larger, patient-based studies.

#### 3.2.6. Nanoparticle Modifications and Functionalization

The strategic modification and functionalization of NPs have significantly augmented their analytical performance. This enables the detection of SCD and thalassemia with enhanced sensitivity and specificity. A range of surface modification and functionalization techniques have been employed to optimize NP performance. Physisorption has been utilized to modify NP surfaces, while surface functionalization with 16-mercaptohexadecanoic acid (MHDA) and sulfobetaine (SB) thiol has enabled the conjugation of NPs with specific biomolecules [40,42]. Additionally, the conjugation of NPs with DNA probes has facilitated the selective detection of target genes associated with SCD and thalassemia. Fibrinogen-conjugated AuNPs and thiol-modified AuNPs have demonstrated exceptional performance in detecting specific SNPs responsible for SCA, achieving impressive detection limits of 12 pM and 1.2 pM, respectively [38,41]. Moreover, the development of composite materials, such as carbon-encapsulated molybdenum disulfide hollow nanorods, has yielded additional enhancements in analytical performance. This underscores the potential of NP modification and functionalization to revolutionize SCD and thalassemia diagnosis [49]. Overall, surface modification and functionalization strategies have played a pivotal role in enhancing the sensitivity, specificity, and performance of NP-based diagnostics. These advancements pave the way for more accurate, targeted, and efficient detection of genetic and protein markers in SCD and thalassemia.

#### 3.2.7. Detection Techniques

The integration of NPs with diverse detection techniques has significantly enhanced the diagnostic capabilities for SCD and thalassemia. Among these, SPRI has proven to be a highly sensitive method for the ultrasensitive detection of point mutations in β-thalassemia using non-amplified genomic DNA [39]. Whereas, another study demonstrated that electrochemical impedance spectroscopy (EIS) is a widely employed analytical technique, enabling sensitive and specific detection of low concentrations of β-thalassemia gene in human serum samples [37]. Using nanogold-based colorimetric detection has shown reliably distinguishing α-globin genes from those containing the Southeast Asian (SEA) deletion responsible for α-thalassemia 1. This approach enables the identification of both carriers and affected individuals, providing a simple and cost-effective diagnostic tool [43]. This study presents a practical nanogold-based assay for genotyping α-thalassemia deletions. It performs well in small-scale testing (*n* = 45) and correlates with conventional methods, but the large-scale clinical validation is still needed [43].

**Figure 4:**
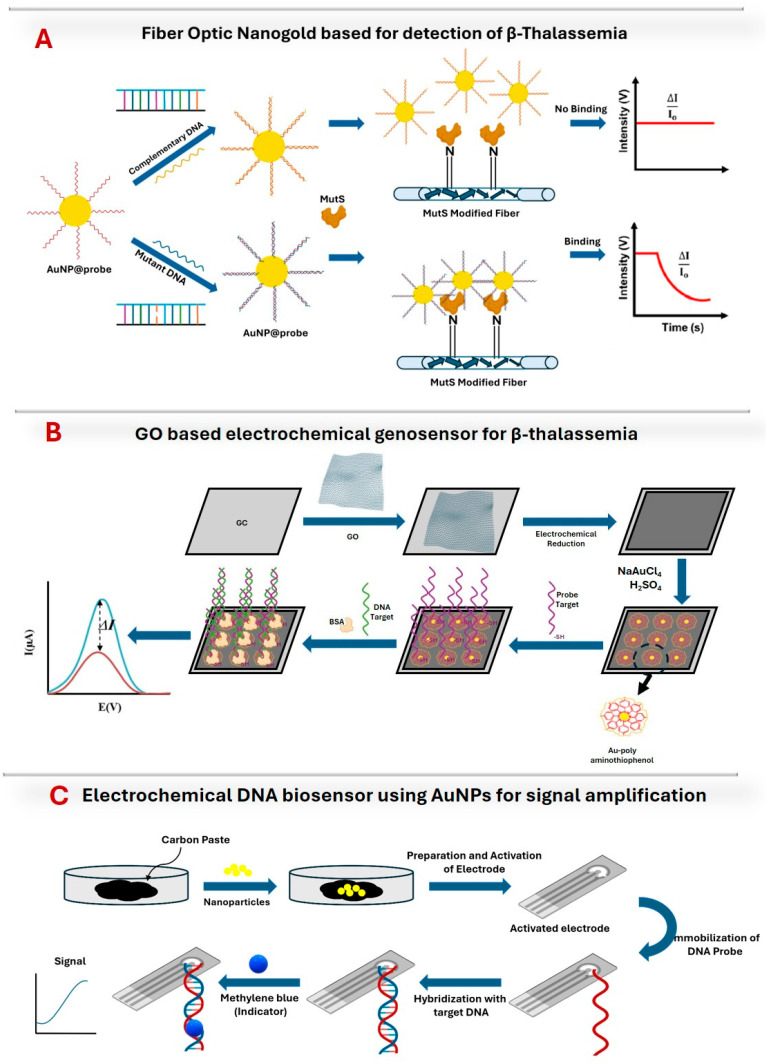
Schematic illustration of genosensor/biosensor-based detection techniques for SCD/thalassemia. (**A**) Fiber optic nanogold-based for detection of β-thalassemia; adapted with modifications from [40]. (**B**) GO-based electrochemical genosensor for β-thalassemia; adapted with modifications from [37]. (**C**) Electrochemical detection of DNA hybridization events using AuNPs for signal amplification; adapted with modifications from [53]. (Abbreviations: AuNPs—gold nanoparticles; MutS—mismatch DNA repair protein; GO—graphene oxide; GC—glassy carbon; NaAuCl_4_—sodium tetrachloroaurate; H_2_SO_4_—sulfuric acid; BSA—bovine serum albumin).

Similarly, a flexible biosensor with ultralow detection limits was developed using AuNP-functionalized graphene-enabled dual-catalytic DNA assembly and colorimetric detection [39]. In another dual-mode biosensing device, AuNPs were combined with MoS_2_@C nanorods to form a stable, high-efficiency self-powered platform for detecting CD122, a thalassemia-associated gene [49]. Similarly, an electrochemical genosensor incorporated AuNPs into a graphene oxide–polymer matrix, resulting in improved electron transfer and precise discrimination of target sequences [37]. Together, these studies underscore how AuNPs have emerged as a highly specific diagnostic tool, exhibiting exceptional performance when combined with these detection techniques. The fusion of NPs with advanced detection techniques like SPRI, EIS, and SERS has revolutionized diagnostics for SCD and thalassemia, offering unparalleled sensitivity and specificity. This multidisciplinary integration holds great promise for translating nanodiagnostics into reliable, real-world clinical tools. Figure 4 summarizes the schematic illustration of different biosensor-based detection techniques for SCD/thalassemia. Figure 4A shows fiber optic nanogold-based detection of β-thalassemia. In this work the fiber-optic sensor is integrated with nanogold-linked probes for detecting cell-free fetal DNA (cfDNA) in maternal plasma. When cfDNA containing the β-thalassemia mutation hybridizes with the immobilized probes, nanogold-conjugated reporter probes bind to the formed duplex. MutS here acts as a molecular mismatch detector which selectively binds to hybridized DNA with β-thalassemia mutations, thus improving the assay’s specificity for prenatal mutation screening. The attachment of nanogold amplifies the optical signal in the fiber optic system, which helps in non-invasive prenatal identification of β-thalassemia [40]. Another GO based electrochemical genosensor for β-thalassemia is represented in Figure 4B. In this biosensor, the carbon electrode is sequentially modified with reduced GO, poly(4-aminothiophenol), and AuNPs to anchor thiolated probe DNA. The hybridization with complementary target DNA alters electron transfer of a redox probe, producing measurable electrochemical signals for thalassemia gene detection [37]. Similarly, Figure 4C depicts electrochemical detection of DNA hybridization events using AuNPs for signal amplification. Here the probe DNA is immobilized on a bimetallic nanocomposite-modified electrode. The complementary target DNA hybridizes to form a double-stranded layer that hinders electron transfer of the redox probe, producing measurable electrochemical changes for sensitive recognition of the thalassemia gene [53].

The current review thus provides valuable insight by systematically collating and comparing LOD values, sample/analyte requirement and functionalization strategies across nanomaterials. This comparative approach reveals the consistent superiority of AuNP-based systems in achieving attomolar sensitivity, the enhanced performance of composite nanostructures (e.g., AuNPs with MoS_2_ or graphdiyne), and the potential of CRISPR-integrated devices for ultrasensitive detection. These insights go beyond summarization to offer practical guidance for prioritizing nanoplatforms with the greatest clinical translation potential.

#### 3.2.8. Clinical Validation

The nanotechnology-based diagnostics for sickle cell disease (SCD) and thalassemia demonstrate impressive analytical sensitivity, however their clinical validation remains limited. Many of the reported detection limits (in the attomolar to femtomolar range) were achieved using synthetic oligonucleotides or spiked samples under controlled laboratory conditions [49,51,54,55]. Although such proof-of-concept studies are valuable for demonstrating analytical potential, their performance may not directly translate to the complexity of clinical samples, where interfering biomolecules, matrix effects, and variable biomarker abundance can significantly affect sensitivity and specificity.

Only a subset of platforms has undergone validation with patient-derived clinical samples (Table 7). For example, AuNP-SPRI assays for β-thalassemia mutations tested 32 patient samples [39], Au@Ag nanoparticle-based SERS assays successfully distinguished β-thalassemia cases (*n* = 8) [50], and quantum dot-based ferritin detection was validated in 16 patient sera [47]. Similarly, nanogold-based colorimetric assays for α-thalassemia included a cohort of 45 patient samples [43]. However, these sample sizes are relatively small, raising concerns about selection bias, limited statistical power, and reproducibility across diverse populations. This underscores the need for large, multi-center studies that not only assess analytical sensitivity but also evaluate performance in real-world clinical settings, including diverse populations and resource-limited contexts.

### 3.3. Practical Implications: Cost, Sample Requirements, and Scalability

Nanotechnology-based diagnostic approaches for SCD and thalassemia exhibit promising analytical performance; however, their practical application depends on multiple translational factors such as cost, sample processing requirements, and scalability. AuNPs are highly sensitive and easy to synthesize but are relatively expensive due to the high price of gold and the need for additional surface modifications (e.g., thiolation, aptamer conjugation). Quantum dots also increase overall cost due to complex processing and surface modifications further add to expenses. This can significantly increase assay costs when scaled to large populations [39,40,45,49,50,51]. By contrast, AgNPs provide a more affordable alternative, offering strong optical properties at lower material cost, though with somewhat reduced sensitivity compared to AuNPs [46,48]. CuO NPs and MNPs are even more cost-effective, being based on inexpensive raw materials and simpler synthesis processes, making them attractive for low-resource settings despite their generally higher LOD values [48,58].

Another important consideration is instrumentation cost. AuNP-based colorimetric assays are relatively low-cost and adaptable to lateral flow or paper-based platforms [43], whereas highly sensitive systems such as SPRI and SERS require sophisticated, high-maintenance instruments, limiting scalability in community or point-of-care settings [39,50]. Quantum dots and upconversion NPs also raise costs due to multi-step synthesis, purification, and functionalization processes, making them less practical for routine screening in low-resource environments [47,56]. Overall, while AuNPs remain the gold standard in sensitivity, more affordable nanoplatforms such as AgNPs, CuO, and MNPs may offer better scalability for population-wide screening, particularly in regions where hemoglobinopathies are highly prevalent but healthcare resources are constrained. Future research should therefore balance sensitivity with cost-efficiency, ensuring that nano-enabled diagnostics can move beyond proof-of-concept toward sustainable clinical implementation.

Furthermore, these platforms differ widely in terms of sample requirements. Most gold and silver NP-based systems are compatible with blood, serum, or isolated DNA, but often require preprocessing steps like DNA extraction or amplification. Some studies, particularly those utilizing quantum dots and upconversion NPs, demand fluorescent signal readouts and specialized instrumentation, limiting field use [30,48,50]. For population-wide screening in low-resource settings, portability and scalability are critical. Gold and silver NP-based colorimetric assays are generally adaptable to lateral flow or paper-based platforms, offering scalable solutions [50,51]. Quantum dot and upconversion NP platforms are less scalable due to cost and reliance on fluorescence imaging equipment. Additionally, biosensors using CuO NPs are easily adaptable to low-cost point-of-care devices due to their colorimetric nature and ease of synthesis [48].

### 3.4. Limitations

While nanotechnology-based diagnostics for SCD and thalassemia show promising analytical performance, several limitations remain. A key concern is the potential for NP aggregation, which can compromise colloidal stability, reduce sensitivity, and lead to inconsistent results during detection [23,25]. Moreover, there is significant variability in NP synthesis methods, including differences in particle size, shape, surface chemistry, and functionalization approaches. Such batch-to-batch variations hinder reproducibility and pose challenges for large-scale clinical translation [24,26]. Another limitation lies in the dependence on sophisticated and expensive instrumentation, such as SPRI and SERS. While these techniques offer high sensitivity, their cost and technical complexity restrict scalability and field-level applicability, particularly in low-resource settings [39,50]. Additionally, the current body of research lacks standardized protocols for NP preparation, assay optimization, and validation, which prevents meaningful cross-comparisons between studies and slows progress toward regulatory approval [30,37]. Most studies were conducted on relatively small sample sizes, highlighting the need for large-scale clinical validation to establish diagnostic accuracy, reliability, and feasibility for routine use [39,47,50]. Addressing these limitations is critical to translating laboratory innovations into practical, cost-effective diagnostic solutions for hemoglobinopathies.

## 4. Conclusions

This systematic review highlights the remarkable progress made in harnessing nanotechnology for the detection of SCD and thalassemia. A comprehensive analysis of 23 studies reveals the exceptional sensitivity and versatility of NP, including gold, silver, quantum dots, and graphene quantum dots. These nanostructures have demonstrated impressive detection capabilities, identifying specific biomarkers and genetic mutations associated with SCD and thalassemia. Recent studies have showcased the exceptional analytical performance of various nanotechnology-based approaches, particularly with AuNPs, in detecting these diseases with high specificity and sensitivity. The highlight is the impressive detection limits achieved by AuNPs-based methods, ranging from 2.6 aM to several pM. This level of sensitivity makes AuNPs a promising material for diagnostic application. The versatility of nanotechnology is also evident in the diverse range of sample types and analytes employed; from blood and serum to DNA, these approaches have demonstrated adaptability and flexibility. Furthermore, strategic modification and functionalization of NPs significantly enhanced their analytical performance. The integration of NPs with various techniques, such as SPRI and EIS, has yielded remarkable results, this tailored approach enables the detection of SCD and thalassemia with improved sensitivity. While these approaches show promise in detection of β-thalassemia, large-scale clinical validation is needed to confirm its utility in diverse patient populations and routine diagnostic settings.

However, despite these promising results, there are few limitations. Most research remains in the early stages, largely confined to laboratory settings and limited proof-of-concept studies, without large-scale clinical validation. Furthermore, some of the most sensitive technologies, such as SPRI and SERS, rely on specialized and expensive equipment, which restricts their applicability in low-resource or field settings. Another critical concern is that many of the included studies were based on small sample sizes, reducing confidence in their clinical generalizability. Addressing these challenges will require standardized protocols, cost-effective platforms, and validation across larger, diverse patient cohorts.

Overall, nanotechnology offers transformative potential for early, sensitive, and cost-effective diagnosis of hemoglobinopathies. Future research should focus on validating these approaches through large-scale clinical studies, simplifying sample preparation, and improving affordability to bridge the gap between laboratory innovation and real-world impact.

## Figures and Tables

**Table 1:** Framework for the systematic review.

Population	Human Suffering from Hemoglobinopathies Like SCD and Thalassemia.
**Intervention**	Use of nanotechnology for detection or quantification of hemoglobinopathy-SCD and thalassemia
**Comparator**	-
**Outcome**	Sensitivity, specificity, limit of detection, limit of quantitation (any of these)

**Table 2:** Databases and other sources used for the literature search.

Type of Database/Literature	Sources
Academic literature	PubMed, Science Direct, Embase
Non-academic search engines	Google Scholar

**Table 3:** Gold nanoparticles for the detection of SCD and thalassemia—using various analytes and techniques.

Nanoparticles	Modifications	Analytes	Techniques	Application	LOD/LOQ	Linear Ranges	Validation	Ref.
Gold	- AuNPs integrated with conductive polymer PAT-Thiolated probe DNA (via self-assembly) - Composite of AuNPs with rGO and PAT	DNA sequences (Human sample)	DPV; EIS	β-thalassemia gene detection in human serum	LOD: 0.035 pM	0.5 pM to 400.0 pM	-	[37]
Gold	- Fibrinogen conjugation - cDNA functionalization - TBA29 assembly	DNA (SNP) related to SCA (Oligonucleotide)	Colorimetry; UV/Vis spectroscopy	Specific SNP detection for SCA	LOD: 12 pM	20–500 pM	-	[38]
Gold	- AuNPs conjugated to biotinylated DNA - Streptavidin attached to the AuNPs	Genomic DNA (Human sample)	SPRI	Diagnosis of β-thalassemia	LOD: 2.6 aM (5 pg/μL)	NA	32 (18 controls, 7 homozygous, 7 heterozygous)	[39]
Gold	DNA probe was physiosorbed to AuNP surface. Surface functionalization with SB thiol and MHDA	Cell-free genomic DNA (Human sample)	FONLISA using FOPPR biosensor	Non-invasive way to diagnose β-thalassemia in fetuses	LOD: 5.2 × 10^−16^ M	NA	-	[40]
Gold	AuNPs are modified with thiol groups, then conjugated with DNA	DNA (gene associated with SCA) (Oligonucleotides)	EW-CRDS	Early disease diagnosis-SCD	LOD: 1.2 pM	NA	-	[41]
Gold	The AuNPs were functionalized with a 30-terminal sulfur of the oligonucleotide. Nano-Au label: The AuNPs were used as a label	Oligonucleotides (point mutation in the β-thalassemia gene)	Piezoelectric method based on the DNA ligase reaction and nano-Au-amplified DNA probes, utilizing QCM for detection	Diagnosis of β-thalassemia	LOD: 2.6 × 10^−9^ mol/L	NA	-	[42]
Gold	Conjugation of AuNPs with SEA-probe to create nanogold SEA-probe	DNA (α-globin gene associated with α-thalassemia 1, SEA deletion) (Human sample)	NP-based colorimetric detection and DNA hybridization techniques	Colorimetric detection of alpha thalassemia	LOD: 200 ug/mL of target DNA by the naked eye	NA	45	[43]
Gold	AuNPs were modified with 3′- or 5′-(alkanethiol) oligonucleotides	DNA (SNP in the β-thalassemia gene) (Oligonucleotides)	Colorimetric analysis	Identification of point mutations in β-thalassemia gene	LOD: 70 fM	0.3 pM to 80 pM	-	[44]
Gold	- Thiolated sDNA modification allowed the AuNPs to hybridize with the tDNA sequence - sDNA-AuNPs were labeled with 6-AFM for fluorescence microscopy imaging	DNA sequence (4 bp deletion in codon 41/42) (Oligonucleotides)	Wireless resonant frequency measurement using a ME DNA-biosensor	Diagnosis of β-thalassemia	LOD: 0.571 pM.	1.0 × 10^−8^ M to 1.0 × 10^−12^ M	-	[45]

NA: not available. Abbreviations: AuNPs—gold nanoparticles; rGO—reduced graphene oxide; PAT—poly(4-aminothiophenol); DPV—differential pulse voltammetry; EIS—electrochemical impedance spectroscopy; TBA29—thrombin-binding aptamer; SNP—single nucleotide polymorphism; SCA—sickle cell anemia; SPRI—surface plasmon resonance imaging; SB—sulfobetaine; MHDA—16-mercaptohexadecanoic acid; FONLISA—fiber optic nanogold-linked sorbent assay; FOPPR—fiber optic particle plasmone resonance; EW-CRDS—enhanced waveguide-cavity ring-down spectroscopy; SEA-probe—thiol-modified DNA probes; 6-AFM—6-amino-fluorescein; ME—magnetoelastic; tDNA—target DNA.

**Table 4:** Other nanoparticles for the detection of SCD and thalassemia—using various analytes and techniques.

Nanoparticles	Modifications	Analytes	Techniques	Application	LOD/LOQ	Linear Range	Validation	Ref.
AgNPs	-	SNPs related to SCA (Oligonucleotides)	Digital imaging (smartphone used to capture images of colorimetric detection and analysis using ImageJ)	SCA Detection: Detection of HbS mutation in the Hbb gene for identifying SCA	LOD: 10^3^ copies per μL of target DNA (HbS) in serum samples	NA	-	[46]
QDs	QDs were modified with avidin, specifically QDs-labeled avidin	Human serum ferritin (Human sample)	- QDs were used as fluorescent labels to detect the binding of the secondary antibody to the primary antibody, allowing for the visualization of ferritin	Thalassemia diagnosis	LOD: 0.27 ng Linear range: 0.27 to 1.1 ng	0.27 to 1.1 ng	16 (4 thalassemia, 10 hepatoma, 2 controls)	[47]
CuO NPs	Interaction of CuO NPs with DNA	Cell-free fetal DNA (Human sample)	Colorimetric nanobiosensor	For prenatal diagnosis of SCA disease	LOD: 0.64 nM Linear range: 2 nM to 12 nM	2 nM to 12 nM	-	[48]

NA: not available. Abbreviations: AgNPs—silver nanoparticles; QDs—quantum dots; CuO—copper oxide; NPs—nanoparticles; SCA—sickle cell anemia

**Table 5:** Nanocomposites for the detection of SCD and thalassemia—using various analytes and techniques.

Nanoparticles	Modifications	Analytes	Techniques	Application	LOD/LOQ	Linear Range	Validation	Ref.
AuNPs and MoS_2_@C	-MoS_2_ NPs encapsulated in carbon to form MoS_2_@C NPs MoS_2_@C NPs modified with PDDA AuNPs conjugated to PDDA-modified MoS_2_@C NPs to form AuNPs/MoS_2_@C NPs	CD122 gene (Human sample)	- EOCV - Colorimetric detection using a smartphone camera	Thalassemia gene detection	- EOCV: LOD: 78.7 aM - Colorimetric: LOD: 58.5 aM.	- EOCV: 0.0001–100 pM - Colorimetric: 0.0001–10,000 Pm	-	[49]
Au@Ag NPs	Sulphhydryl-modified capture probe bound to Au@Ag NPs via Ag-S bonding	MicroRNA-210 (miR-210) (Human sample)	- SERS - RT-qPCR	Early and rapid screening of β-thalassemia	LOD: 5.13 fM	10 fM–1.0 nM	8 (4 β-thalassemia, 4 controls)	[50]
AuNPs/GDY nanocomposite	- AuNPs GDY nanosheets - AuNPs/GDY nanocomposite functionalized with a substrate material to create a bioelectrode - Bioelectrode was modified with glucose oxidase and a CRISPR/Cas12a-ordered concatemeric DNA probe to create a self-powered biosensing system	CD142 gene (Human sample)	- OCP - Visual signal detection (using colorimetric assay with TMB and HRP) - CRISPR/Cas12a-based biosensing	Detection of thalassemia in human serum samples.	LOD: 48.1 aM of CD142 DNA sequence.	0.0001–100 pM	-	[51]
AuNPs and graphene oxide NPs	- AuNPs - 4-Aminothiophenol (4-ATP) to form a self-assembled monolayer (SAM) - Glutaraldehyde - Graphene oxide NPs—ammonium functionalization	DNA (SCA mutation) (Oligonucleotide)	EIS	Diagnosing and screening SCA	LOD: 40 pM	40–1000 pM	-	[52]
Platinum (Pt) and AgNP	Modified with carbon paste to form MCPE. Additionally, the AgNPs were also modified with Pt to form bimetallic nanocomposite electrode	PCR-amplified β-globin gene sequence (Human sample)	EIS and LSV	Detection of β-thalassemia	LOD: 470.0 pg/μL	NA	-	[53]
HgTe nanostructures Gold	HgTe nanostructures were used as the matrix for SALDI-MS analysis. AuNPs were functionalized with thiol-modified capture ODNs	Single- and double-stranded oligodeoxynucleotides (related to SCD) (Oligonucleotide)	SALDI-MS Analysis	Analyzing a SNP that determines the fate of the valine residue in the β-globin chain of sickle cell megaloblasts.	LOD: 0.05 μM	0.1–1.0 μM	-	[54]
Ag/Pt bimetallic nanoclusters	Tri-plex-Ag/PtNCs and LNA modified X-shaped DNA probe	DNA (single-nucleotide variant related to β-thalassemia) (Oligonucleotide)	EIS, CV, and SWV	Detection of single-nucleotide variants (SNVs) related to β-thalassemia.	LOD: 0.8 fM	NA	-	[55]
NaYF_4_:Yb^3+^, Er^3+^ NPs	Silica coating: A thin layer of SiO2 was deposited on the NPs. DNA was conjugated to the silica-coated NPs using a CNBr activation method	DNA (Oligonucleotide)	- Photoluminescence measurements - Spectro fluorometry	Detection of point mutation associated with SCD	LOD: 120 fM	NA	-	[56]
AuNPs/GDY nanocomposite	AuNPs were functionalized with graphdiyne (GDY) to form AuNPs/GDY nanocomposites. These were then used to modify the surface of a flexible carbon cloth (CP) electrode	CD122 gene (Human sample)	- EIS, CV, and LSV to detect the target CD122 gene - Colorimetric assay to detect the target CD122 gene	Detection of the CD122 gene associated with thalassemia in human serum samples	LOD: 36.3 aM (electrochemical mode) and 12.1 aM (colorimetric mode)	0.0001–10,000 pM	-	[57]

NA: Not available. Abbreviations: EOCV—electrochemical open circuit voltage; MoS_2_@C—carbon-encapsulated molybdenum disulfide hollow nanorods; Au@Ag NPs—gold–silver bimetallic nanoparticles; GDY—graphdiyne; 4-ATP—4-Aminothiophenol; SAM—self-assembled monolayer; Pt—platinum (Pt); SCA—sickle cell anemia; PDDA—poly(diallyldimethylammonium chloride); SERS—surface enhanced Raman scattering; OCP—open circuit potential; MCPE—modified carbon paste electrode; EIS—electrochemical impedance spectroscopy; LSV—linear sweep voltammetry; HgTe—mercury telluride; SALDI-MS—surface-assisted laser desorption/ionization mass spectrometry; CV—cyclic voltammetry; SWV—square wave voltammetry; SNVs—single-nucleotide variants; CNBr—cyanogen bromide; tri-plex-Ag/PtNCs—triplex DNA-templated Ag/Pt bimetallic nanoclusters; LNA—locked nucleic acid.

**Table 6:** Detection techniques for SCD and thalassemia.

Technique	Advantages	Limitations	Sensitivity	Ref.
EIS	High sensitivity, label-free detection	Requires specialized equipment, limited multiplexing capabilities	LOD: 0.035 pM Linear range: 0.5 pM to 400.0 pM	[37]
SPRI	Real-time detection	Requires specialized equipment, limited multiplexing capabilities	LOD: 2.6 aM (5 pg/μL) Linear range: NA	[39]
Fluorescence spectroscopy	Multiplexing capabilities	Requires fluorescent labels, photobleaching concerns	LOD: 120 fM Linear range: NA	[56]
Colorimetric detection	Low cost, easy to perform, label-free detection	Limited sensitivity, subjective interpretation.	LOD: 0.64 nM Linear range: 2 nM to 12 nM	[48]
SERS	High sensitivity, multiplexing capabilities	Requires specialized equipment, limited reproducibility	LOQ: 5.13 fM Linear range: 10 fM–1.0 nM	[50]
QCM	Real-time detection	Requires specialized equipment, limited multiplexing capabilities	LOD: 1.2 pM Linear range: NA	[41]
LSV and CV	Provides information on redox reactions and simple equipment required	Time-consuming and require specialized equipment	LOD: 470.0 pg/μL Linear range: NA	[53]
MS	Sensitivity and specificity, multiplexing capabilities, and real-time detection	Requires specialized equipment and Time consuming	LOQ 0.4 nmol/L Linear range: NA	[58]

NA: not available. Abbreviations: EIS—electrochemical impedance spectroscopy; SPRI—surface plasmon resonance imaging; SERS—surface enhanced Raman scattering; QCM—quartz crystal microbalance; LSV—linear sweep voltammetry; CV—cyclic voltammetry; MS—mass spectrometry.

**Table 7:** Validation context of reported LODs.

Nanoplatform/Technique	Target (Gene/Marker)	Sample Type	Validation Context	Sample Size	Ref.
AuNPs-SPRI	β-thalassemia point mutations	Genomic DNA	Patient-derived samples	32 (18 controls, 7 homozygous, 7 heterozygous)	[39]
Au@Ag NPs + SERS	miR-210	Erythrocytes	Patient-derived samples	8 (4 β-thalassemia, 4 controls)	[50]
AuNP nanogold–colorimetric assay	α-thalassemia (SEA deletion)	DNA	Patient-derived samples	45	[43]
Quantum dots	Serum ferritin	Human serum	Patient-derived samples	16 (4 thalassemia, 10 hepatoma, 2 controls)	[47]
Boron-doped graphene QDs	Hematin	Erythrocytes	Patient-derived samples	7 (5 controls, 2 SCD)	[59]

## Data Availability

The authors confirm that the data supporting the findings of this study are available within the article. Raw data that support the findings of this study are available from the corresponding author, upon reasonable request.

## Abbreviations

The following abbreviations are used in this manuscript:

4-ATP	4-Aminothiophenol
6-AFM	6-Amino-Fluorescein
AgNPs	Silver Nanoparticles
Au@Ag NPs	Gold–Silver Bimetallic Nanoparticles
AuNPs	Gold Nanoparticles
BSA	Bovine Serum Albumin
CNBr	Cyanogen Bromide
CuO	Copper Oxide
CV	Cyclic Voltammetry
DPV	Differential Pulse Voltammetry
EIS	Electrochemical Impedance Spectroscopy
EOCV	Electrochemical Open Circuit Voltage
EW-CRDS	Enhanced Waveguide-Cavity Ring-Down Spectroscopy
FONLISA	Fiber Optic Nanogold-Linked Sorbent Assay
FOPPR	Fiber Optic Particle Plasmon Resonance
GC	Glassy Carbon
GDY	Graphdiyne
GOD	Glucose Oxidase
H_2_O_2_	Hydrogen Peroxide
H_2_SO_4_	Sulfuric Acid
HPLC	High-Performance Liquid Chromatography
HgTe	Mercury Telluride
LNA	Locked Nucleic Acid
LOD	Limit of Detection
LSV	Linear Sweep Voltammetry
MCPE	Modified Carbon Paste Electrode
ME	Magnetoelastic
MHDA	16-Mercaptohexadecanoic Acid
MoS_2_	Molybdenum Disulfide
MoS_2_@C	Carbon-Encapsulated Molybdenum Disulfide Hollow Nanorods
MNPs	Magnetic Nanoparticles
MS	Mass Spectrometry
NaAuCl_4_	Sodium Tetrachloroaurate
NaCl	Sodium Chloride
NMs	Nanomaterials
NPs	Nanoparticles
OCP	Open Circuit Potential
PAT	Poly(4-Aminothiophenol)
PDDA	Poly(Diallyldimethylammonium Chloride)
PRISMA	Preferred Reporting Items for Systematic Reviews and Meta-Analyses
Pt	Platinum
QCM	Quartz Crystal Microbalance
QDs	Quantum Dots
rGO	Reduced Graphene Oxide
RT-qPCR	Reverse Transcription Quantitative Polymerase Chain Reaction
SALDI-MS	Surface-Assisted Laser Desorption/Ionization Mass Spectrometry
SAM	Self-Assembled Monolayer
SB	Sulfobetaine
SCA	Sickle Cell Anemia
SCD	Sickle Cell Disease
SEA	Southeast Asian
SEA-probe	Thiol-Modified DNA Probes
SERS	Surface-Enhanced Raman Scattering
SNP	Single Nucleotide Polymorphism
SNVs	Single-Nucleotide Variants
SPRI	Surface Plasmon Resonance Imaging
SWV	Square Wave Voltammetry
TBA29	Thrombin-Binding Aptamer (29-mer)
TMB	Tetramethylbenzidine
tDNA	Target DNA
tri-plex-Ag/PtNCs	Triplex DNA-Templated Ag/Pt Bimetallic Nanoclusters
